# Editorial: Psychological changes through the lifespan: Ageing and psychosocial-related variables

**DOI:** 10.3389/fpsyg.2022.948329

**Published:** 2022-07-14

**Authors:** Carol Holland, Qian Xiong, Esperanza Navarro-Pardo

**Affiliations:** ^1^Centre for Ageing Research, Lancaster University, Lancaster, United Kingdom; ^2^Department of Developmental and Educational Psychology, University of Valencia, Valencia, Spain

**Keywords:** aging, psychosocial variables, cognition, psychological changes, lifespan, dementia, healthy aging

This series of papers examines aspects of aging cognition in both healthy older adults and those diagnosed with dementias, and although few in number, gives an important overview of some of the key issues in examining the relationships between aging and psychosocial variables, including social cognition. The special issue includes five papers which cover: goal related decision making (Ito et al.); trust in cooperative behavior (Telga and Lupianez); impact of anxiety on self-perceived cognitive difficulties (Spalding et al.); factors associated with depression in Wuhan, China (Liu et al.); and neurophysiological functional connectivity related to apathy in people living with dementia (Altunkaya et al.).

The first two papers both examined the role of cognitive demand, respectively in decision making and trust in cooperative behavior (Ito et al.; Telga and Lupianez). Both papers proposed that the tendency toward more automatic processing and greater difficulty inhibiting learned responses in older adults may lead to more habitual and perseverative decisions, where learning occurred only from feedback, as opposed to continual effortful monitoring (Ito et al.), or in more use of stereotypes or heuristics in making decisions about who to trust or who is more likely to be co-operative (Telga and Lupianez). Ito et al. confirmed previous studies (e.g., Eppinger et al., 2013) in that older adults were more habitual and younger people more goal-oriented. However, these authors did not find that participants relied on expected age-related stereotypes (where older people are often judged as more trustworthy) and neither did older participants rely on the less cognitively demanding heuristic of stereotyping. Indeed, all participants adopted an individuation processing style in which individual behavior was used to learn who might behave cooperatively. Telga and Lupianez suggest that in this study, older adults' tendency toward more automatic processing using heuristics may be overridden by their motivation to achieve harmonious relationships, following Carstensen et al.'s (1999) theory of socioemotional selectivity. While such motivations were unlikely to influence the methodology of the Ito et al. study, both studies also manipulated possible financial motivations.

The role of socioemotional selectivity and regulation was also an important feature of the article focusing on the impact of trait anxiety on everyday cognitive difficulties (Spalding et al.); it examined the role of age as a moderator of the relationship between anxiety and self-observed cognitive difficulties, emphasizing the difference between cognitive anxiety (e.g., worry) and somatic anxiety (e.g., rapid heart rate). The authors proposed that the effect of anxiety on cognition would increase with age, especially for cognitive anxiety, relating this to the Strengths and Vulnerabilities Integration model (SAVI; Charles, 2010). The SAVI model proposes that both strengths and vulnerabilities of cognitive processing change with increasing age. A strength of cognitive processing in older age is inhibition and re-evaluation of negative thoughts and experiences to accommodate for more positive memories and emotional wellbeing (positivity effect, Carstensen and Mikels, 2005), while a vulnerability is that limited cognitive resources are needed to regulate both emotions and the effects of anxiety. The authors proposed that if older adults prioritize emotion processing over other cognitive functions, this may result in apparent deficits in those other functions. They also proposed that, alternatively if older adults have less cognitive control, such as when there are executive function changes, then normal emotion regulation may fail, leading to greater impact of anxiety in older adults. This was supported in the study: age moderated the effect of cognitive anxiety on most cognitive functions, so that even when there had been no significant direct effect of anxiety on cognition, age increased the effect size in the moderation analyses to a significant level. The fact that this moderation was only consistent for cognitive anxiety and not for somatic anxiety is important, supporting the SAVI model. This has possible implications for the importance of supporting older adults experiencing anxiety or trauma.

The Research Topic aimed to include articles examining the impact of a range of external and internal factors on the psychological processes of outcomes. The article by Liu et al. examined a range of factors related to depression in a community in Wuhan, China. The multidimensional nature of the predictors of depression was a real strength of this study, showing that physical frailty, educational level, cognitive function, functional independence (Activities of Daily Living, ADLs), as well as age itself, were significant predictors in the model.

The final paper examined another psychosocial process that specifically impacts some people living with dementia, that of apathy. Altunkaya et al. compared people with Alzheimer's disease, subcortical ischemic vascular disease (SIVD) and cognitively unimpaired older adults in terms of functional connectivity changes in a brain imaging study. They found that the group with SIVD had the highest level of apathy, and that factors such as depression, dementia stage, and volume of white matter hyperintensities (WMH, an indicator of damage to white matter connective pathways) were predictors of apathy. The analysis of different resting state networks focused on difficulties with initiating action, an indicator of apathy. The understanding of apathy in people living with dementia is important given the significant impact inertia can have on positive behaviors such as social engagement or on activities of daily living, leading to increased carer burden and potential institutionalization.

The timing of the studies in relation to the pandemic was an important factor in some of the studies, offering a unique set of circumstances to examine, but also potentially affecting generalizability. For example, the research reported by Liu et al. was conducted during the months of September to October 2020, at the height of the COVID-19 pandemic; for this reason, the timing may have influenced the results. The authors found that both living situation and social support were among the set of important predictors of depression in this cross-sectional analysis. Timing was also a feature for the Ito et al. study discussed above. One of the main objectives of this study was to validate the decision-making task methodology in an online format for older adults, due to the impossibility of seeing older adults in person during the COVID-19 lockdowns. Previous validations of the online version have not included older adults and this could be a problem, given potential differences in computer confidence, access and literacy. The authors replicated previous findings on decision-making style and learning, and also identified some of the advantages of online studies, such as the possibility of obtaining a larger and more diverse sample and overcoming physical accessibility issues of studies where participants have to come to a laboratory, but also disadvantages, such as limitation to those with a computer and reasonable confidence in its use, or lack of supervision of their attention to the task by a researcher.

In summary, the roles of cognitive demand and cognitive control, emotional processing, and socio-emotional selectivity, are threads that weave through these studies, with important implications for attention to and interventions for the predictors of depression, apathy and anxiety highlighted, including in both healthy older adults and those with diagnoses of dementia.

## Author contributions

All authors listed have made a substantial, direct, and intellectual contribution to the work and approved it for publication.

## Conflict of interest

The authors declare that the research was conducted in the absence of any commercial or financial relationships that could be construed as a potential conflict of interest.

## Publisher's note

All claims expressed in this article are solely those of the authors and do not necessarily represent those of their affiliated organizations, or those of the publisher, the editors and the reviewers. Any product that may be evaluated in this article, or claim that may be made by its manufacturer, is not guaranteed or endorsed by the publisher.
